# Phenotype-Genotype Correlation in Morquio A Syndrome: Protocol for a Meta-Analysis

**DOI:** 10.2196/56649

**Published:** 2024-11-14

**Authors:** Lorena Diaz-Ordoñez, Paola Andrea Duque-Cordoba, Daniel Andrés Nieva-Posso, Wilmar Saldarriaga, Juan David Gutierrez-Medina, Harry Pachajoa

**Affiliations:** 1 Department of Basic Medical Sciences Center for Research on Congenital Anomalies and Rare Diseases (CIACER) Universidad Icesi Cali Colombia; 2 School of Basic Sciences Universidad del Valle Cali Colombia; 3 UROGIV Research Group School of Medicine Universidad del Valle Cali Colombia; 4 Hospital Universitario del Valle Cali Colombia; 5 Centro de Investigaciones Clinicas Fundación Valle del Lili Cali Colombia

**Keywords:** Morquio A syndrome, genotype-phenotype associations, rare diseases, scoping review, Mucopolysaccharidosis type IV, meta-analysis, genotype, GALNS gene, N-acetylgalactosamine-6-sulfatase, pathophysiology, laboratories, mutations

## Abstract

**Background:**

Mucopolysaccharidosis type IVA (MPS IVA), also known as Morquio A syndrome, is a rare lysosomal storage disease characterized by autosomal recessive inheritance of mutations in the N-acetylgalactosamine-6-sulfatase (*GALNS*) gene. This leads to a deficiency of the GALNS enzyme, causing the accumulation of glycosaminoglycans in tissues. Morquio A syndrome primarily affects the skeletal system and joints but can also impact various organs, resulting in symptoms such as hearing and vision loss, respiratory issues, spinal cord compression, heart diseases, and hepatomegaly. The genotype-phenotype relationship is diverse, with studies highlighting variants associated with classic, nonclassic, or intermediate phenotypes. Understanding these genetic factors is crucial for predicting disease prognosis and tailoring effective treatment strategies for individuals with Morquio A syndrome.

**Objective:**

The aim of this meta-analysis is to comprehend the relationship between the severity of the phenotype and the genotype of patients with MPS IVA, considering factors such as the type of variant and its location in the different domains of the protein.

**Methods:**

This meta-analysis will include articles featuring participants of all genders and age groups who have a molecular diagnosis of MPS IVA and a description of the phenotype. Literature published in English, Spanish, and Portuguese will be considered. Exclusion criteria will encompass studies lacking full-text availability and those involving patients with an MPS IVA diagnosis but without phenotype information. The databases to be searched include PubMed, MEDLINE, ScienceDirect, and Scopus. The screening of literature, paper selection, and data extraction will involve 2 independent reviewers, who will conduct the process blindly. In the event of disagreements between the 2 reviewers at any stage, resolution will be sought through discussion or with the involvement of an additional reviewer. The final selection of manuscripts will be based on consensus. The results of the review will be presented using descriptive statistics, and the information will be organized in either diagrammatic or tabular formats, following the guidelines provided by the Joanna Briggs Institute. Genotype-phenotype relationships will be analyzed using IBM SPSS Statistics, using chi-square tests, Fisher exact tests, and regression analysis to interpret the data.

**Results:**

A literature search conducted in January 2024 produced 760 results. The review is expected to be completed by the end of 2024.

**Conclusions:**

This meta-analysis will gather and analyze information on the phenotype-genotype relationship in patients diagnosed with MPS IVA. The data collection and resulting analyses will make a substantial contribution to understanding the underlying mechanism of the disease, enabling the prediction of the syndrome’s progression and severity.

**International Registered Report Identifier (IRRID):**

DERR1-10.2196/56649

## Introduction

Mucopolysaccharidosis type IVA (MPS IVA), also known as Morquio A syndrome, is a rare lysosomal storage disease characterized by autosomal recessive inheritance. It originates from a defect in the N-acetylgalactosamine-6-sulfatase (GALNS) enzyme, resulting from mutations in the *GALNS* gene [1].

The deficiency of the GALNS enzyme leads to the accumulation of glycosaminoglycans, such as chondroitin-6-sulfate and keratan sulfate, in various tissues and organs. This primarily causes symptoms in the skeletal system and joints, but other clinical manifestations can also occur, including hearing and vision loss, respiratory diseases, spinal cord compression, heart diseases, and hepatomegaly [2].

The phenotypic spectrum of the disease is broad, ranging from attenuated forms (nonclassic) with late-onset clinical manifestations and slow progression to severe forms (classic) with early-onset clinical manifestations [1].

The *GALNS* gene is located on chromosome 16 region q24.3 and consists of 14 exons [3]. It encodes 13 transcripts, with *NM_000512.5* as the reference transcript, spanning a length of 2344 base pairs, which codes for a protein with 522 amino acids [4].

The protein resulting from the *GALNS* gene, with UniProt accession number P34059, forms a homodimer, where each monomer in its x-ray crystallographic structure presents 3 domains: an N-terminal domain containing the active site (28-379), a second domain with antiparallel B chains (380-481), and a C-terminal domain (482-510) that complements the active site where a calcium ligand binds. Each monomer contains 2 N-linked glycosylation sites at asparagine 204 and asparagine 423, and 3 disulfide bonds (308-419, 489-518, and 501-507), along with an unpaired cysteine (164) [5].

The genotype-phenotype relationship has garnered increasing interest in predicting disease prognosis based on genetic findings. It requires more in-depth analysis, considering factors such as the type of variant, its location in the protein domain, and its effect on the tertiary structure. For instance, Cárdenas et al [6] found that variants associated with the classic phenotype affected highly conserved protein regions. However, statistically significant results were not obtained due to limitations in sample size [6].

In contrast, Zanetti et al [7] analyzed 314 homozygous individuals, identifying 135 variants. Of these, 103 were associated with a classic phenotype, 19 with a nonclassic phenotype, 2 with an intermediate phenotype, and 11 with conflicts in phenotype classification. Discordance between phenotype classification and patient genotype may be influenced by differences in the age of diagnosis [7].

Tomatsu et al [8] found that among missense variants, the most common variants were associated with a classic phenotype (63 cases) or an attenuated phenotype (30 cases). Eight nonsense mutations and 2 large deletions were linked to severe phenotypes; 16 small deletions were associated with severe phenotypes, with 4 being undefined; 4 insertions were linked to severe phenotypes, with 1 associated with an attenuated phenotype; and 8 splice-site mutations were associated with severe phenotypes, while 1 was linked to an attenuated phenotype [8]. Concurrently, specific variants have been associated with distinct phenotypes, such as c.1156 C>T, present in 6 homozygous individuals with a classic phenotype, and c.761 A>G, associated with an intermediate phenotype in 2 homozygous patients [9].

Although the relationship between specific variants and the severity of MPS IVA has been previously demonstrated, there are certain limitations that could lead to discrepancies in the analyzed data. These limitations include the use of enzyme replacement therapy, variations in the age at which clinical characteristics are evaluated, and the interaction between variants in compound heterozygous patients. Hence, this meta-analysis proposes to determine the relationship between different types of genetic variants in the *GALNS* gene and the severity of the phenotype in patients with MPS IVA.

## Methods

This meta-analysis proposal is based on the parameters stipulated by the PRISMA (Preferred Reporting Items for Systematic Reviews and Meta-Analyses) 2020 guidelines [10] and the Cochrane Collaboration [11].

### Eligibility Criteria

Studies will be included that involve participants of any gender and age, with a molecular diagnosis of MPS IVA, along with a description and/or classification of the clinical phenotype.

### Inclusion Criteria

The included studies must meet the following criteria:

- Molecular diagnosis of MPS IVA
- Classification regarding the severity of the phenotype or information on clinical manifestations that enables classification, according to guidelines or protocols endorsed by genetics societies

### Exclusion Criteria

The exclusion criteria are as follows:

- Individuals with a diagnosis other than MPS IVA
- Unavailable full-text articles
- Articles with patients with MPS IVA but without a molecular diagnosis

### Types Sources

This meta-analysis will encompass a broad range of study designs, incorporating both experimental and quasi-experimental methodologies. Experimental designs, such as randomized controlled trials, nonrandomized controlled trials, before-and-after studies, and interrupted time-series studies, will be included. Additionally, observational studies, including both prospective and retrospective cohort studies, case-control studies, and analytical cross-sectional studies, will be considered for inclusion.

Furthermore, various observational study designs, such as case series, individual case reports, and descriptive cross-sectional studies, will also be considered for inclusion in this review. Qualitative studies will be considered as well.

### Search Strategy

The search strategy has been designed to identify published studies exclusively in Spanish, English, and Portuguese regardless of the publication date. Key terms from article titles and abstracts, as well as index terms, have been used to formulate a comprehensive search strategy for various databases, including MEDLINE, Web of Science, the Cochrane Library, ScienceDirect, PubMed, and Scopus.

The Medical Subject Headings (MeSH) keywords used for the database search are as follows: (GALNS OR “galactosamine-6-sulfatase” OR “Mucopolysaccharidosis IV”) AND (“genetic variants” OR “genetic variations” OR “mutations”) AND (“clinical” OR “phenotype”).

The search will be conducted by a single reviewer, who will perform the search in the specified databases, adhering to the MeSH keywords outlined above.

### Study Selection

The identified bibliography will be uploaded to the Covidence platform (Veritas Health Innovation Ltd), duplicates will be removed, and 3 reviewers will screen the literature based on the title and abstract. Subsequently, the full text will be analyzed to verify compliance with the inclusion criteria. Exclusion reasons will be categorized and reported in the systematic review. Any disagreements will be resolved by an additional reviewer or through consensus among the reviewers. The results will be presented in the final systematic review.

### Data Extraction

A data extraction instrument was created. A total of 2 researchers will extract the data from each record. Extraction fields include the following: the country where the study was conducted, year of publication, data characterization (physiopathology, diagnostic, epidemiology, management or treatment, and complications), study design, medical history, clinical diagnosis, diagnostic test and results, and clinical interventions. Any disagreements that arise between the reviewers at each stage of the selection process will be resolved through discussion or with an additional reviewer.

### Data Analysis and Presentation

A descriptive tabulation of the necessary information will be conducted to establish the genotype-phenotype relationship in patients with MPS IVA, encompassing epidemiology, physiopathology, and genetic diagnosis. Data will be analyzed using IBM SPSS Statistics software. The genotype-phenotype association will be assessed using chi-square or Fisher exact tests and/or regression analysis. The entirety of gathered knowledge will be summarized and discussed, taking into consideration the limitations of the review.

Compound heterozygous variants will not be considered in the genotype-phenotype analysis due to the inherent complexity of interactions between these variants. However, they will be individually described and discussed, aiming to establish a precedent for future research and publications in the field.

Furthermore, in the event of discrepancies in the phenotypes of homozygous patients carrying the same variant, the authors of the original publications will be contacted to investigate potential factors that may contribute to this variability, such as age at diagnosis and the use of enzyme replacement therapy. Each finding will be discussed in detail.

### Reference Searches

Snowballing or citation tracking criteria will be applied to identify significant articles pertinent to the research topic. This process will involve utilizing the reference list of a paper or citations of a paper by other articles to uncover additional manuscripts relevant to the study’s subject. Once potential new manuscripts and citations are identified, a backward snowballing search will be conducted by scrutinizing the reference list and excluding papers that do not meet the basic inclusion criteria. Subsequently, previously examined papers from the list will be eliminated. If a paper aligns with the inclusion criteria, potential new manuscripts will be pinpointed by reviewing the reference list of the included paper [12]. Subsequently, forward snowballing will be conducted, identifying new papers from the reference list of included papers, utilizing a method like backward snowballing.

### Expected Findings

This exploratory review aims to categorize the phenotypic severity of patients with MPS IVA and associate these phenotypes with specific genotypes. By mapping each pathogenic variant onto the GALNS protein structure, the analysis will evaluate how these variants in different domains and regions of the protein influence disease severity. This approach will help identify patterns of pathogenic variants associated with varying levels of clinical severity, providing key insights into how the location and type of variant affect GALNS functionality and the overall severity of MPS IVA. The findings will offer a deeper understanding of genotype-phenotype relationships, with potential to guide future diagnostic and therapeutic approaches.

## Results

**Figure 1:**
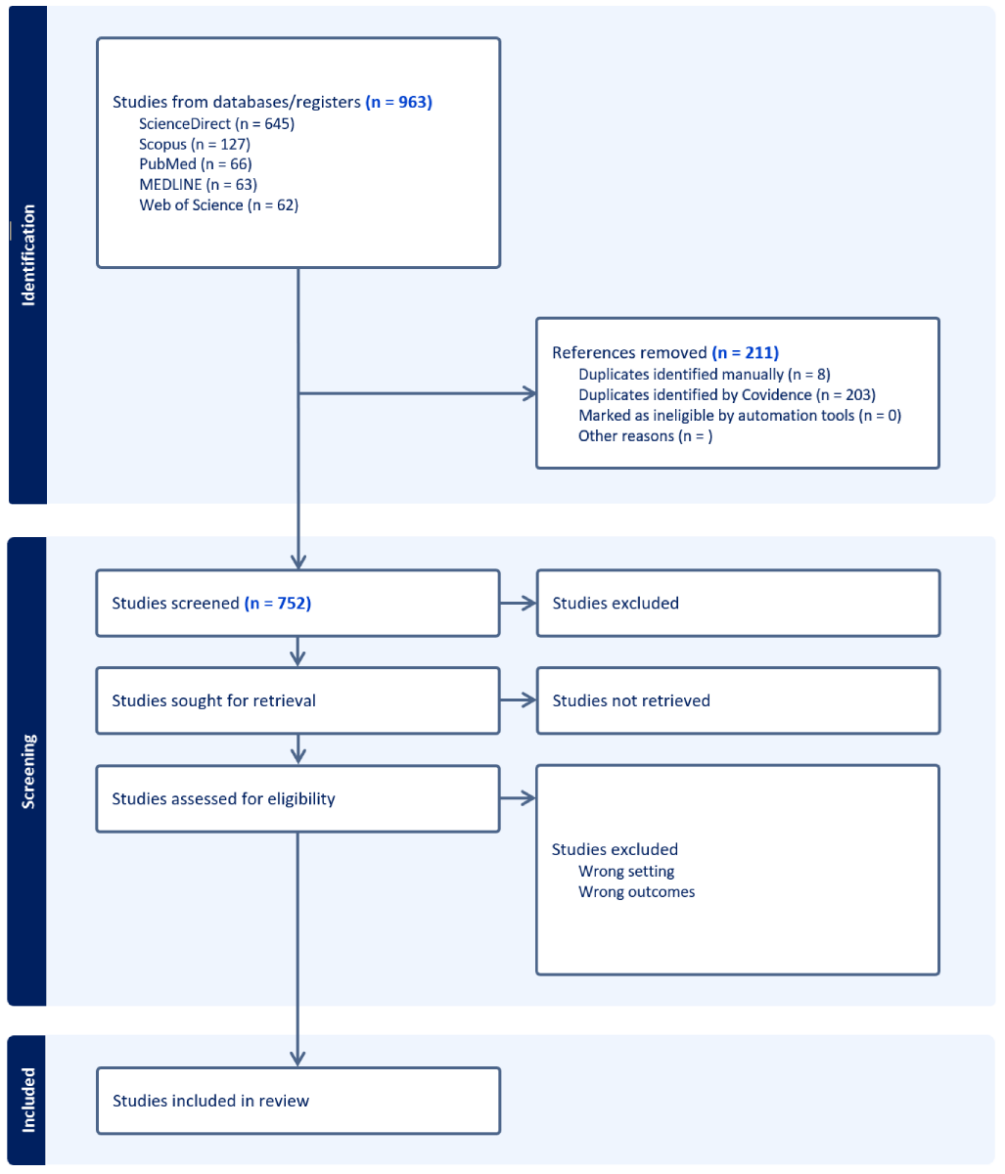
Flowchart of the literature selection process.

In January 2024, a comprehensive literature search was conducted in PubMed, MEDLINE, ScienceDirect, and Scopus, identifying 752 articles after duplicate removal (Figure 1). The review will be completed by the end of 2024, with all relevant manuscripts managed through Covidence software to streamline the systematic review process.

## Discussion

### Summary of the Main Findings

The meta-analysis is expected to demonstrate the relationship between the type of genetic variation and its location in the GALNS protein and the severity of the phenotype in patients with MPS IVA.

### Contrasting Literature

The relationship between *GALNS* variants and their impact on pathophysiology, severity, and treatment opportunities is a well-studied topic. Several investigations have explored the genotype-phenotype relationship, such as the study by Zanetti et al [7], which included 314 patients with 135 different variants. Of these variants, 103 were associated with a severe phenotype, 19 with a nonsevere phenotype, 2 with an intermediate phenotype, and 11 with conflicting clinical phenotypes. According to the authors, the inconsistency in phenotype-genotype classification could be attributed to the heterogeneity of the disease and the varying ages of the patients at diagnosis [7].

Additionally, studies such as Yi et al [13] found 82 patients with a severe phenotype, 14 with an intermediate phenotype, and 12 with an attenuated phenotype. The classification included both homozygous and compound heterozygous patients and was based on the type of variants and their location in the protein [13].

Furthermore, some identified variants have even led to the identification of new clinical variations that have led to the reporting of new cases, such as the one identified by Ge et al [14] that establishes a relationship between neurogenic bladder and MPS IV.

Among the findings on the correlation between genotype or genetic variations in mutations and the patient’s phenotypic presentation, studies such as Tomatsu et al [15] stand out. The authors proposed that some genetic variations in conserved regions directly affect the enzyme’s functional capacity and lead to specific phenotypic alterations that require a more in-depth intervention process in patients with MPS IVA [15]. Other studies, such as Jezela-Stanek et al [16], found that phenotype-genotype assessment and correlation have been shown to predict aspects related to anthropometric measurements and associated complications.

On the other hand, this meta-analysis presents several limitations, including variations in phenotype severity that are independent of genotype. For instance, enzyme replacement therapy, designed to mitigate disease progression, may influence the outcomes. Additionally, age differences are a significant factor, particularly in patients diagnosed at early stages, where clinical characteristics may not yet be fully developed. Furthermore, in compound heterozygous patients, it is challenging to establish a precise relationship between genotype and phenotype, as the final characteristics will be affected by the interaction of both variants.

### Conclusions

Understanding the relationship between variant types, protein mutation location, and patient phenotypes will enhance our comprehension of which protein sites and types of variants have the greatest impact on the pathophysiology of MPS IVA. This knowledge will not only aid in predicting disease severity in new cases but also facilitate more precise monitoring of disease progression, serving as a marker for complications associated with Morquio A syndrome. These insights will have broader implications for guiding medical interventions, allowing for greater precision and effectiveness in treatment strategies.

## Abbreviations

GALNS: N-acetylgalactosamine-6-sulfatase
MeSH: Medical Subject Headings
MPS IVA: mucopolysaccharidosis type IVA
PRISMA: Preferred Reporting Items for Systematic Reviews and Meta-Analyses

## Data Availability

All data generated or analyzed during this study are included in the published paper.
